# Analytical and Theoretical Studies of Antioxidant Properties of Chosen Anthocyanins; A Structure-Dependent Relationships

**DOI:** 10.3390/ijms23105432

**Published:** 2022-05-12

**Authors:** Anita Dudek, Maciej Spiegel, Paulina Strugała-Danak, Janina Gabrielska

**Affiliations:** 1 Department of Physics and Biophysics, Wrocław University of Environmental and Life Sciences, C. K. Norwida 25, 50-375 Wrocław, Poland; 113767@student.upwr.edu.pl (A.D.); janina.gabrielska@upwr.edu.pl (J.G.); 2 Department of Pharmacognosy and Herbal Medicines, Wroclaw Medical University, Borowska 211A, 50-556 Wrocław, Poland

**Keywords:** anthocyanins, liposome, oxidation, computational studies

## Abstract

The relationship between the structure and the antiradical and antioxidant activities of three anthocyanidins, namely peonidin, petunidin, and delphinidin, and their glucosides was investigated in this study. The ability of anthocyanins to scavenge free radicals was determined using DPPH^●^ assay, whereas the inhibition of peroxidation in liposomes in relation to a model membrane that imitated the composition of a lipid membrane in tumor cells was specified using the fluorimetric method. To explore this issue at the atomistic level, density functional theory studies were applied. It was shown that glycosides performed better than anthocyanidins in protecting membranes against oxidation. The highest redox potential was demonstrated by anthocyanidins with the highest number of hydroxyl groups in the B ring in the order as follows: (Dp > Pt > Pn), and the same relationship was proven for their glucosides. The majority of the compounds studied here proved to be better antioxidants than ascorbic acid. They showed consistent electrodonating properties and though the *f*-HAT mechanism became more feasible with each consecutive deprotonation. Glycosylation did not have a direct impact on reactivity, apart from peonidin and petunidin in the study of which it was found that this process was responsible for lifting off steric hindrance between B and C rings and rendering certain pathways more feasible. Kinetic and molecular dynamics are essential to properly describe the membrane’s lipid oxidation.

## 1. Introduction

Nowadays, there is an increasing interest in natural compounds found in plants and their potential health benefits. Particular attention is given to compounds that play a role as natural antioxidants and have a protective function against oxidative stress in the human body. Oxidative stress is a process which is generated by the production and accumulation of reactive oxygen species (ROS) in cells and tissues, as well as the ability of the biological system to detoxify these reactive molecules. ROS can interact with lipids, proteins and nucleic acids and cause damage to cells and tissues. This condition of imbalance may lead to cardiovascular and respiratory diseases, cancer, autoimmune, and neurolo-gical diseases [1].

Anthocyanins, more specifically flavonoids, are polyphenolic compounds which are characterized by a C6–C3–C3 carbon skeleton. They are water-soluble plant pigments present in almost all parts of plants, including leaves, flowers, and fruits. Besides being pigments, they are also responsible for performing many other functions in plants, as well as other flavonoid compounds. Anthocyanins have the ability to absorb UV radiation, prevent photo-oxidation and photodegradation, protect plants against pathogens, pests, cold stress, and provide them with drought resistance [2,3]. In addition to their significant role in plants, recently, anthocyanins have increasingly been at the center of scientific interest due to their potential health benefits. It has been shown that the consumption of products rich in dietary anthocyanins can prevent the development of so-called civilization diseases, e.g., cardiovascular diseases and diabetes, by increasing glycogen content in cells, regulating glucose uptake and transport, and managing the amount of insulin released in obesity through increasing energy consumption and inhibiting lipid absorption [4]. Moreover, anthocyanins exhibit antimicrobial activity against a wide range of microorganisms, improve visual health, and also have anti-cancer effects against many types of cancer, including breast, colon, prostate, liver, and lung cancers [5,6,7]. Many of these beneficial effects are related to the antioxidant and anti-inflammatory properties of anthocyanins.

Although there are many techniques for determining the antioxidant activity of anthocyanins, each of them should be appropriate to the type of compound, its structure or stability. Many of the methods are based primarily on spectrophotometry, fluorimetry, or calorimetry. They involve two main mechanisms, namely the formal hydrogen atom donator (*f*-HAT) mechanism and the single-electron transfer (SET). In the *f*-HAT mechanism, the free radical removes a hydrogen atom from the antioxidant (AH+), transforming the free radical into a more stable product. In the SET mechanism, the antioxidant (AH+) donates an electron to the free radical, reducing the oxidized intermediate to a stable form [8]. Some of the further methods to determine the antioxidant capacity of anthocyanins in vitro are: the DPPH^●^, ORAC (oxygen radical absorbance capacity), TRAP (total peroxyl radical trapping antioxidant parameter) and FRAP (ferric reducing antioxidant power) assays [8,9]. These and other methods were designed among others to provide us with information on the antioxidant activity of anthocyanins determined by their ability to chelate metal ions such as Cu(II) or Fe(III) and to neutralize ROS in a reaction with a stable and coloured radical as in the DPPH^●^ assays. Furthermore, fluorimetric methods such as ORAC are based on reactions with a specific probe located in the oxidized membrane and the observation of changes in its fluorescence under the influence of free radicals, also in the presence of an antioxidant. Using these methods, one can determine the actual antioxidant activity of anthocyanins, which is one of the most important steps in determining their overall biological activity. 

The common aglycon forms, anthocyanidins, found in plants are as follows: cyanidin, delphinidin, peonidin, petunidin, malvidin, and pelargonidin, but may also be present in the form of glycosides with attached sugar moiety most often at C3 position in C ring or less frequently at the C5 or C7 position in A ring. The sugar substituent might be acylated by aromatic or aliphatic acids. Biological activity, including antioxidant properties and free radical scavenging of anthocyanins, is ensured, among others, by the presence of a different number of hydroxyl groups located in specific positions. Apart from hydroxylation and glycosylation, the structure of anthocyanins can also be modified by methylation and acylation, thus influencing the antioxidant activity. Together, these structure modifications may alter the biological properties of anthocyanins, as well as their colour, stability, bioavailability, and interaction with other biomolecules, such as lipids or proteins [9]. 

The primary goal of this study was to determine the in vitro antioxidant activity of three anthocyanidins, namely peonidin, petunidin, and delphinidin, as well as their C3-glucosides: peonidin 3-*O*-glucoside, petunidin 3-*O*-glucoside and delphinidin 3-*O*-glucoside (Table 1). These substances were selected to demonstrate the importance of their presence in the B ring, their arrangement, and the presence of a glycosidic substituent at the C3-fixed position and, finally, in determining the antioxidant activity on model lipid membranes. We also conducted theoretical research based on the density functional theory framework in order to find answers to these questions which are widely used nowadays to study the reactivity of various chemical structures including antioxidants [10]. We proposed to look into anthocyanins (AA) and their ability to capture a stable free radical in the DPPH^●^ test to see if they could inhibit peroxidation of liposomes. A physicochemical factor, AAPH^●^, was used to induce lipid peroxidation in a model membrane that mimicked the lipid composition of tumor cells (MM). By applying theoretical chemistry, we were able to elucidate experimentally observed activities at the atomistic level and effectively examine both the electro- and thermochemistry properties of these compounds which relate directly to the antioxidative activity. The proposed research may contribute to the description of the way in which anthocyanins protect against lipid peroxidation, in particular in regard to the lipid bilayer of tumor cell membranes, and shed light on the potential of their possible application in medicine.


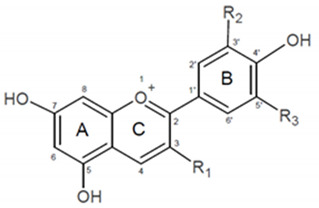


## 2. Results and Discussion

### 2.1. Antioxidant Activity and Free-Radical Scavenging Activity

**Figure 1:**
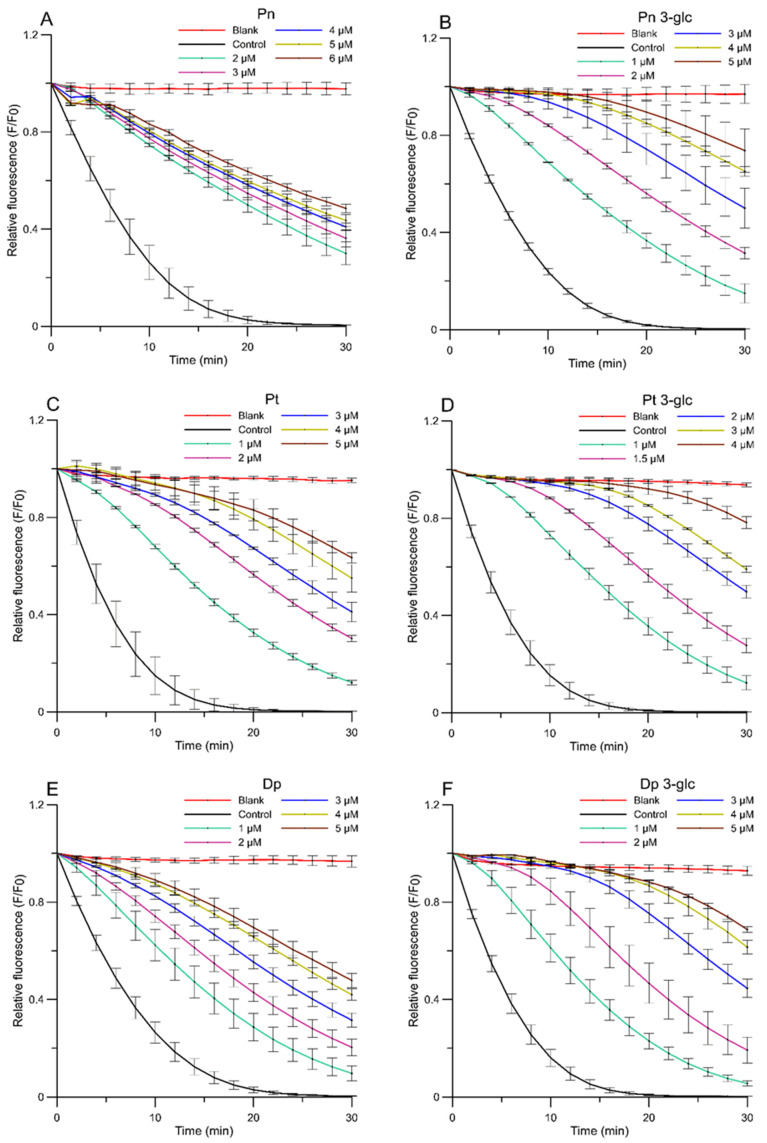
Relative fluorescence intensity of DPH-PA probe as a function of time of oxidation a lipid mimic membrane for AAPH^●^ radicals in the presence of anthocyanins at selected concentrations. (**A**) peonidin, (**B**) peonidin 3-*O*-glucoside, (**C**) petunidin, (**D**) petunidin 3-*O*-glucoside, (**E**) delphinidin, (**F**) delphinidin 3-*O*-glucoside. The relative change in fluorescence intensity F/F_0_ is a measure of the degree of lipid peroxidation. (F_0_—fluorescence in the control sample (oxidized by AAPH^●^ without anthocyanins), F—fluore-scence of samples in the presence of anthocyanins). Blank sample—not oxidized by AAPH^●^ and without anthocyanins.

Two antioxidant assays, namely the liposome oxidation assay and the DPPH^●^ free-radical scavenging test, were determined in vitro using fluorimetric and spectrophotometric methods. The antioxidant activity of AA was studied using fluorimetric method based on thermal decomposition in the AAPH compound causing oxidation in the liposome membrane. The alkyl radicals induced by AAPH^●^ caused oxidation in the DPH-PA probe (3-[p-(6-phenyl)-1,3,5-hexatrienyl]propionic acid), reducing its fluorescence. The results are presented as kinetic curves of changes in the relative fluorescence intensity of the DPH-PA probe at the presence of different concentrations of AA during 30 min of reaction (Figure 1). As seen in Figure 1, the relative fluorescence for each anthocyanin concentration decreased in line with the oxidation time, indicating that the degree of inhibition of lipid oxidation increased. Based on the oxidation kinetics diagram, the percentage of oxidation inhibition after 30 min was calculated. Afterwards, the dependence of the percentage of oxidation inhibition on the concentration of the AA was determined (linear relationships not shown) and, on this basis, the IC_50_ parameter was estimated. While interpreting the kinetic curve, it may be concluded that all AA protected the lipid membranes against peroxidation induced by AAPH^●^ significantly but there were great differences between compounds. At the highest concentration used, the glycosides were more active (Figure 1B,D,F) and inhibited the oxidation process better than anthocyanidins, while Pn had the lowest activity (Figure 1A). Pt 3-glc (Figure 1D) showed the best protective effect on liposome membranes during the 30 min reaction time and in the first phase of the reaction, at the highest concentrations, it almost entirely prevented lipid oxidation. Respectively, for all compounds during the further course of the reaction, when the amount of AA began to run out, the fluorescence intensity decreased and was practically linear.

**Figure 2:**
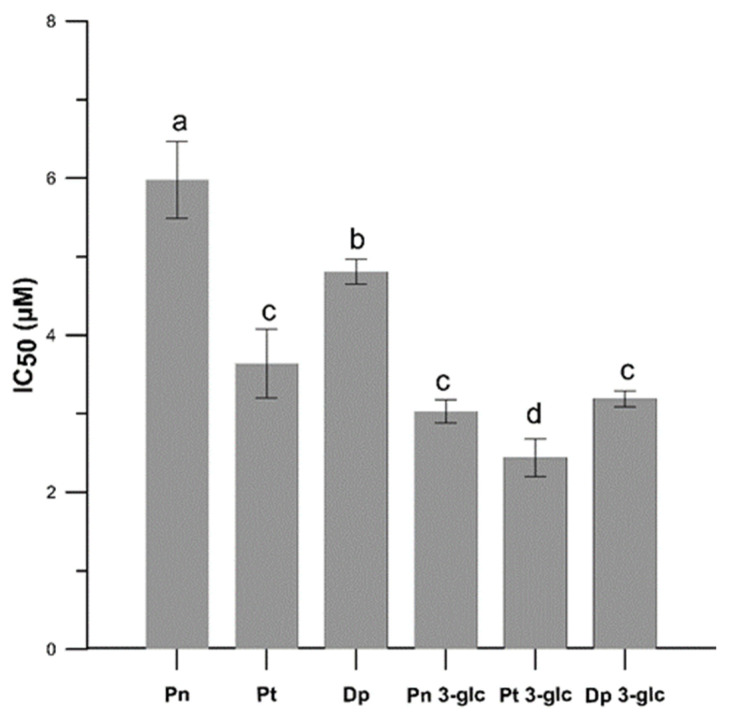
The antioxidant activity (IC_50_) for Pn, Pt, Dp, Pn 3-glc, Pt 3-glc and Dp 3-glc into lipid mimic membrane. Membrane oxidation was induced with AAPH^●^ compound. IC_50_ values are the averages of three independent measurements ± SD. Different letters (a–d) indicate significant differences (*p* < 0.05).

Based on the IC_50_ parameter (Figure 2), which represents the concentration of the studied compounds that inhibits 50% of lipid peroxidation process, the Pt 3-glc showed the best antioxidant activity (IC_50_ = 2.44 ± 0.24 µM), whereas Pn 3-glc (IC_50_ = 3.03 ± 0.15 µM), Dp 3-glc (IC_50_ = 3.19 ± 0.10 µM), and Pt (IC_50_ = 3.64 ± 0.44) also appeared to perform well as antioxidants. To compare, ascorbic acid was also tested as a commonly used antioxidant. All of the results obtained indicated that the tested anthocyanins showed much better antioxidant properties in comparison with ascorbic acid (IC_50_ = 114.64 ± 14.61 µM) and protected the model membrane against oxidation more effectively than ascorbic acid, approximately 36–47 times stronger in the case of glycosides and c.a. 19–32 times stronger in the case of anthocyanidins. Although differences between the antioxidant activity of anthocyanidins and anthocyanins were apparent in most cases, there was hardly any correlation between the number of -OH groups in the molecules and the ability to inhibit lipid oxidation. This is confirmed by the fact that the highest activity was observed for Pt 3-glc with two hydroxyl groups in the ring B of molecule and not, as it could be expected, for Dp, which contains 3 -OH groups in the B ring. This pattern was observed for Pn because it had the lowest number of hydroxyl groups and the weakest antioxidant properties.

Previous studies suggested a significant relationship between the amount of hydroxyl groups and antioxidant properties. It was demonstrated that delphinidin-3-*O*-rutoside had higher antioxidant activity than cyanidin 3-*O*-glucoside against Fe(II)-induced liposome oxidation, which was explained by the presence of more -OH groups in the B ring of delphinidin, which, in turn, enhanced its antioxidant activity [11]. Another study showed that antioxidant activity against Fe(II)-induced lipid peroxidation improved with an increase in the number of hydroxyl substituents present in the B ring of the anthocyanidins, whereas the substitution of hydroxyl groups with methoxy groups -OCH_3_ decreased the antioxidant potential of anthocyanidins [12]. On the other hand, one investigation reported that cyanidin and cyanidin 3-*O*-glucoside had the highest inhibitory effect on the oxidation of lipoprotein (LDL) induced by copper (II), whereas delphinidin had only intermediate effectiveness [13]. In the same study, a similar relationship between the presence of a sugar substituent and a decrease in antioxidant capacity was observed for the 3-O glucosides malvidin and cyanidin, which were respectively 50% and 90% less active than the corresponding anthocyanidins. Some authors pointed out that in the case of malvidin, not only the presence of the 3 -OH group in the C-ring, but especially the dihydroxyl structure in the B-ring, was crucial for maintaining high antioxidant activity. Therefore, glycosylation of this anthocyanin at 3-position may not significantly reduce the antioxidant activity as much as diminished number of -OH group in the ring B [14]. In a study conducted by Cyboran-Mikołajczyk et al. [15], it was found that the antioxidant properties towards POPC (1-palmitoyl-2-oleoylphosphatidylcholine) containing membranes were closely related to the presence of -OH groups in the structure of the compound and it was demonstrated that the presence of an additional sugar substituent significantly decreased the antioxidant properties of cyanidins and their monosaccharides. The authors suggested that the presence of disaccharides or two sugar molecules alters the specific structure of the compound, reduces its interaction with the membrane, and thus may limit the availability of -OH groups involved in the scavenging of free radicals induced by AAPH^●^. The results we obtained, nevertheless, showed that glucosides, especially Pt 3-*O*-glc, showed a better protective effect towards lipids MM. The reason for this may be the greater hydrophilicity of the glycosides compared to the anthocyanidins, which allows for easier incorporation into the surface lipid bilayer and thus better protection against free radicals. 

**Figure 3:**
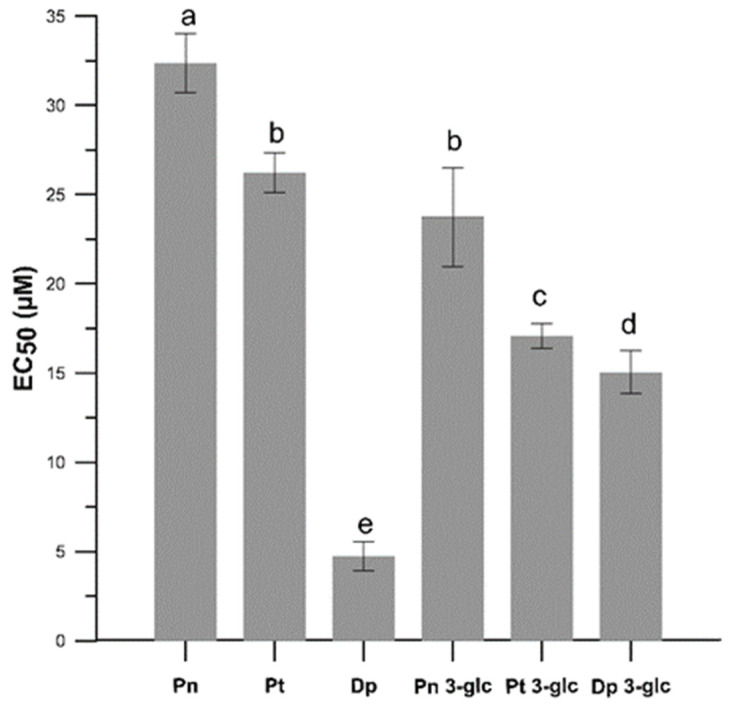
The antiradical activity (EC_50_) in the neutralization process of the DPPH^●^ free radicals caused by Pn, Pt, Dp, Pn 3-glc, Pt 3-glc and Dp 3-glc. EC_50_ values are the averages of three independent measurements ± SD. Different letters (a–e) indicate significant differences (*p* < 0.05).

We also tested the anti-radical activity of the AA using a DPPH^●^ test and determined the effective concentration of the EC_50_ parameter (Figure 3). In this experiment, differences between the anti-free radical activity of anthocyanidin and the glucosides forms were also observed, and the glycosides exhibited greater anti free-radical activities than the anthocyanidins, except for Dp which was more active than Dp 3-*O*-glc. The results obtained in free-radical scavenging assay indicated that Dp and its glucoside possessed the highest activity, followed by petunidin and peonidin derivatives. The lowest effectiveness was demonstrated for Pn and Pn 3-glu. Comparing the EC_50_ parameter for ascorbic acid EC_50_ = 18.5 ± 0.006 µM (data from our previous research [16]), it can be stated that the anthocyanins studied here, with the exception of Dp, Dp 3-*O*-glc, and Pt 3-*O*-glc, are less effective scavengers of DPPH^●^. 

The differences in free radical scavenging activity obtained in the DPPH^●^ test of anthocyanidins and anthocyanins (Figure 3) may be related to the number and position of hydroxyl and methoxy groups, as well as the presence of a sugar substitute. The results showed a correlation between the number of -OH groups in the B ring and the free radical scavenging abilities of both anthocyanidins (Dp >> Pt > Pn) and anthocyanins (Dp 3-glc > Pt 3-glc > Pn 3-glc). Pn with only one -OH group in the B ring at the 4′ position showed the weakest action and Dp, whereas this which had two additional groups at the 3′ and 5′ positions in this ring showed the strongest action. Replacing one of the hydroxyl groups with a methoxy group as in petunidine also reduced the effect of the antiradical. Such a connection was also observed by Fukumato and Mazza, who showed that Dp having three hydroxy groups in the B ring was more effective in scavenging DPPH^●^ than cyanidin with hydroxy groups only in the 3′ and 4′ positions [17]. Some research showed that anthocyanidins without the *O*-diphenyl structure, such as Pn and Pt, were both less effective anti-radical against DPPH^●^ than Dp and this is also confirmed by our study. Results conducted by Rahman et al. [18] showed antioxidant activity towards superoxide and peroxynitrite radicals also demonstrated a relation between the presence of hydroxy and methoxy groups and antioxidant capacity. This study has shown that the activity decreased with *O*-methylation in the B ring. Therefore, the compounds in the order which shows the highest activity were as follows: delphinidin > petunidin > malvidin. It can be also seen that the sugar substituent reduced the antioxidant activity of the compounds, which may be explained by the fact that substitution of glucose at position 3 in A ring deprived the compounds of one of the hydroxy groups and thus reduced the chances of effective radical scavenging. It is suggested that substitution of the hydroxy group at position 3 in the C with another substituent, e.g., a sugar substituent, may lead to a reduction in the chelating capacity of the metal ion and additionally influence the structure and planarity of the molecule by changing its conjugation ability and the angle of torsion with the B and C rings, which is correlated with the overall antioxidant properties [19,20,21]. According to literature, the antioxidant potential of anthocyanidins is generally higher than that of their corresponding glycosides, and consequently, the sugar substituent may significantly reduce the antioxidant properties [22,23]. However, some studies showed the contrary effect and indicated increased antioxidant properties of the sugar substituted forms. Huang et al. [24] demonstrated that malvidin glycosides improved antioxidant activities compared to the corresponding anthocyanidins. Furthermore, in the same study, differences were observed between malvidin-3-*O*-glucoside and malvidin 3-*O*-galactoside. The compound with the sugar residue in the form of glucose was a more potent antioxidant. According to Jim et al. [25], this enhanced antioxidant activity of glycosides can be attributed to the electron-donor action of the 3-bulky sugar group and, more specifically, the sugar substituent or the hydroxyl substituent are both hydrogen bond acceptors and donors. 

On the basis of the analysis of the experimental data, differences in the activity relationships among both anthocyanidins and their glucoside derivatives should be highlighted. They show that the ability to free radical scavenging/antioxidant activity under oxidative stress conditions of lipid membranes cannot be explained only by the redox potential of AA, but also by another mechanism of lipid membrane protection. It is planned to continue experimental studies in order to fully unravel this issue.

### 2.2. Theoretical Investigation

#### 2.2.1. Dissociations Constants and Deprotonation Routes

As stressed earlier, for a proper representation of the experimental data, it is essential to consider all species that would exist within water solvent in a relevant concentration. This is particularly important in the case when, for example, a radical is efficiently scavenged by hydrogen atom related channels only, whereas at given conditions entirely deprotonated particles are present. Without considering acid–base equilibria and obtaining from them molar fractions, improper conclusions can be drawn from thermodynamic and kinetic results. Therefore, p*K*_a_ values and molar fractions (^M^*f*) of each acid–base species were assessed at physiological pH (Table 2). The graphical visualization of deprotonation pathways and the distribution diagrams are presented in Supplementary Materials (Figure S1).

The data indicate that neutral, monoanionic, and di–anionic forms are the prevalent ones. Cation of peonidin, as well as tri-anions of delphinidin and petunidin, are present in non-negligible populations (^M^*f* > 0.1%), but their molar fractions are particularly low when compared to the previously mentioned species.

The shift in the dissociation constants is observed as the result of glycosylation, although in a non-uniform manner. While p*K*_a1_ increases in all cases, the next either raise or drop. This phenomenon is particularly fascinating, bearing in mind that the C3 hydroxyl group does not undergo deprotonation as the first one in any case. However, at the same time, proton affinity values change when that position is substituted by glucose (Table S1). Nonetheless, the process of glycosylation is not expected to directly influence the electronic structure of the flavylium ring though the resonance, nor result in a formation of hydrogen bonds among hydroxyls of the saccharide with those of the anthocyanidins due to a considerable distance between them. 

#### 2.2.2. Electronic Structure Properties

To elucidate the electronic structure in regard to glycosylation pattern, studies at the atomistic level are necessary. The first step was to assess intrinsic reactivity indices which represent solid foundations for the discussion on these issues. Ionization potentials (IP) and bond dissociation enthalpies (BDE) are sufficient to represent, in general, the reactivity of isolated antioxidants owing to the following reasons: (1) a potent radical scavenger preferably act as an electron donator rather than an acceptor, leaving electron affinity as an irrelevant determinant; (2) proton affinity, an initial step of the SPLET mechanism, does not need to be established for acid–base species already involved in the study; (3) proton transfer, the second part of the ETPT mechanism, can be omitted because the first step drives the reaction further. The validity of these approximations, namely restricting to BDE and IP, is confirmed by results obtained for studies drawing from the QM-ORSA protocol which applies them and is constantly reported to yield theoretical values with errors similar to those arising from experiments [26,27,28]. 

As evidenced from the established indices (Table 3), BDE values corroborate with the pattern presented earlier for PAs. No significant variation between the feasibility of the *f*-HAT mechanism shall be observed between pentyl ethanoate and water. However, this assumption concerns just peonidin and petunidin 3-*O*-glucoside, because they are the only present in cationic form in the polar solvent. On the other hand, deprotonation can be linked with the increasing reactivity of the compound, both BDE and IP drops.

The differences in BDEs of the same sites between the different aglycones, the different glycosides, or the anthocyanidin and its corresponding anthocyanin are generally marginal. Homolytic fission from the C3 hydroxyl group is most feasible among nearly all compounds having yet undissociated it. Contrarily, in the case of their glucosides, this is typically C4′. Inspecting the dihedral angles between B and C rings, hereafter represented by Φ, of pentyl ethanoate species, to assess properties originating from the structure, not solvent, it was found that while for delphinidin and its glucoside they are nearly the same, 21.1° and 20.1°, in the case of peonidin Φ equals 33.0°, while in the case of peonidin 3-*O*-glucoside it was found to be 19.1°. Even greater values were noted for petunidin (Φ = 36.8°), and again lower for its glycoside (Φ = 22.3°). The role of dihedral angle, so important for electron delocalization [29], manifests here and explains why upon glycosylation of the latter two anthocyanidins not only do most of the BDEs somehow lower, but the reaction sites also shift. C4′ position is then particularly feasible because the opportunity to form stable hydroquinone-like motif is granted. 

Ma et al. investigated four anthocyanins from purple potato [30] and though the authors restricted to the cationic forms in water, these have been found to have lower BDE values at relevant C4′ positions, and higher ionization potential, when compared to petunidin 3-*O*-glucoside. These species are glycosylated in two positions, namely C3 and C5, but the observed effect is most likely to stem from the vicinity of methoxy groups, whose role was already investigated for phenolic acids [31] and appears from our results.

#### 2.2.3. Electron and Hydrogen Donating Ability Map of Antioxidants

To identify species which exhibit plausible antiradical activities and allow for a comparison between them or other reference compounds, an eH-DAMA (electron and hydrogen donating ability map of antioxidants) can be constructed. Its main goal is to assess the feasibility of two major mechanisms antioxidants utilized to scavenge free radicals: formal hydrogen atom transfer and single electron transfer. The *f*-HAT mechanism is represented by the bond dissociation energy, whereas SET can be either described by ionization potential or electron affinity, if an electron is donated to radical or accepted from it, respectively. Several previous papers have already evidenced that potent antioxidants generally prefer to scavenge reactive species by providing them with electrons [32,33]. However, reverse pathways can also be a valid way of neutralizing carbon-centered radicals of nucleophilic character [34] or to convert O_2_^−•^ to ^3^O_2_ [35]. Nonetheless, for species with particularly low IP values such as polyanions, Gibbs free energy does not allow for the proper assessment of single electron transfer proclivity because they are found in the inverted region of the Marcus parabola, observed as the reappearing increase in an activation energy of reaction. [36]. Electrophilicity index represents a much more feasible determinant of that process for while it also relies on IP, it does so in a nonlinear fashion, with the shape of this dependency resembling the Marcus parabola [37]. 

**Figure 4:**
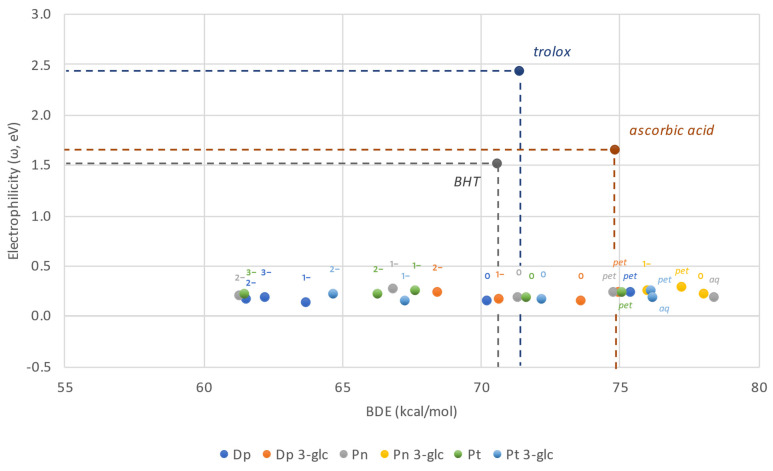
Electron and Hydrogen Donating Ability Map of Antioxidants plotted for anthocyanidins and anthocyanins. Neutral forms of Trolox, BHT and ascorbic acid are presented for comparison purposes.

Bearing in mind that lower values of ω correspond to the efficient scavenging in the course of SET mechanism, and that lower BDE shall account for the greater participation of *f*-HAT mechanism, species located at the bottom left of the map are particularly appealing antioxidants. Figure 4 shows the eH-DAMA constructed for the investigated compounds and additionally Trolox, ascorbic acid, and BHT in their neutral forms as reference substances. 

All the anthocyanidins and anthocyanins are predicted to have similar electron–donor capabilities, much better than the reference compounds, however they differ among themselves in H–donating potential. Based on the data collected, it can be stated that while ω remains nearly constant regardless of the ionic state, *f*-HAT appears to be more feasible with each consecutive deprotonation. As a result, the majority of the neutral and anionic structures distinguish as much better antioxidants than ascorbic acid. However, not all of them are better than Trolox or BHT. This is valid solely for, at least, the monoanions. These outcomes are generally favorable considering that neutral- and singly deprotonated species represent the major fractions found in the solution. On the other hand, cationic species, regardless of whether water or pentyl ethanoate is being discussed, are at maximum as effective in hydrogen donation as ascorbic acid, but many of them are even worse. Peonidin-3*-O*-glucoside stands out in that collation as any of its forms has a BDE value lower than the previously referenced antioxidant.

#### 2.2.4. Thermochemistry

The initial results obtained from intrinsic reactivity indices and eH-DAMA account for the promising radical scavenging potential of the anthocyanidins and anthocyanins. Nonetheless, only by combining experimental and theoretical studies this question can be answered properly. This was achieved by estimating Gibbs free energies of *f*-HAT and SET reactions between the studied species and DPPH^•^ in water. While the influence of a change in entropy can be generally neglected in the case of small radicals modelled in the course of typical computational studies, it is relevant in the bulk species like that one.

Unfortunately, several issues on modeling the APPH^●^ assay in silico need to be addressed, which disallowed that. The first one is the actual species antioxidants scavenge. Bearing in mind that several are produced in the course of the reaction, particularly alkoxy and peroxyl radicals of AAPH^●^, as well as radicalized forms of DPH-PA, a single target cannot be pointed. Water-soluble APPH converts itself into radicals in the aqueous environment, which means they need to reach unsaturated parts of DPH-PA deep in the lipid bilayer for the change in fluorescence to be observed. The profile of the curve representing time ~ relative fluorescence was reported not to differ from the one obtained for other radical-initiator, AMVN^●^, which in contrast localize in-between lipids [38]. Although APPH^•^ decomposition was initiated just before the reaction, the rates at which radicals react are high, reaching 1.2 × 10^3^ M^−1^s^−1^ for the reaction between ^●^OOH and polyunsaturated fatty acid, or even above the diffusion limit (>10^8^ M^−1^s^−1^) as evidenced for the most reactive ^●^OH [28,39,40]. 

It was already stressed there are discrepancies with the data of other scientists, suggesting a permeability into the lipid bilayer might be of significant merit. Deprotonation is a spontaneous reaction with a great rate constant. Therefore, compounds actually reach acid–base equilibria before diffusion takes place. Only the neutral ones are capable to penetrate the bilayer, putting an emphasis on their molar fractions. However, while deprotonated species are neutral, the consequence of proton detachment is the formation of a localized negative charge and the dipole momenta (Table S2). In the case of delphinidin and peonidin, glycosylation results in the charge being more equilibrated along the structure, whereas petunidin 3-*O*-glucoside is greater than the aglycone.

With the presented drawbacks accounting for the observed anti-APPH^●^ activity, it is evidenced that the process is much more complicated than one would assume. To describe it properly, two important aspects must be covered and combined. The first one is investigations on the motion corresponding to the entering of all the studied species into lipid bilayer, and so molecular dynamic simulations. The other is reaction kinetics, which allow to assess at what rate radicals are scavenged. Nonetheless, these are out of the scope of this paper and instead shall be thoroughly presented in a forthcoming manuscript. 

The DPPH^●^ outcomes, in contrast, can be satisfyingly explained with quantum mechanical studies due to the simplicity of the assay. The thermochemical calculations of *f*-HAT (Table 4) feasibility reflect the pattern found for BDEs and PAs underlining previous elaborations on the topic. In accordance with the estimated molar fractions, neutral and monoanionic species are generally the most abundant and they shall majorly participate in the reactivity.

Although DPPH^●^ is thoroughly reported to be scavenged by hydrogen acceptance, it can also be reduced to the stable anionic form. However, with electron transfer theory elucidating the factors responsible for determining SET activation energy, it cannot be stated that the lowest Gibbs free energy shall also represent the most reactive species. Strongly negative values may fall in the inverse region of the Marcus parabola, where activation energy increases. For this reason, reaction barriers have been computed instead and the reaction rates are provided (Table 5). As expected, activation energies drop with consecutive deprotonation, and for dianions of delphinidin and peonidin, as well as tri-anion of delphinidin, are so low that the reaction is only diffusion limited. This particular observation is very plausible recalling that dianion delphinidin represents ~15% of the solution’s composition. Delphinidin shall participate most readily in SET reactions, while the activities of peonidin and petunidin are comparable. However, it can be seen that glycosylation increases the activation energies of delphinidin and peonidin, while lowering them for petunidin.

While the DPPH^●^ assay is a good reference to estimate *f*-HAT and SET viability as validated by the experiment, from the biological point of view, the goal of antioxidants is to scavenge certain radicals, particularly hydroperoxides. Having the computational chemistry results (consult Supplementary Materials), it can be noticed that hydrogen atom donation from the studied compounds to ^●^OH, ^●^OCH_3_, and ^●^SH is nearly always very exergonic. On the other hand, ^●^OOH is not neutralized so easily, but it looks like the electron donation is the main mechanism antioxidants undergo to scavenge it. Moreover, it is in agreement with the eH-DAMA map plotted for DPPH^●^ that with each consecutive deprotonation, the Gibbs free energy of hydrogen atom transfer drops down. Concluding, the investigated structures are truly attributed with a significant antiradical potential.

## 3. Materials and Methods

### 3.1. Materials

Peonidin, petunidin, delphinidin, and their glucosides peonidin 3-*O*-glucoside (Pn 3-glc), petunidin 3-*O*-glucoside (Pt 3-glc), and delphinidin 3-*O*-glucoside (Dp 3-glc) were purchased from Extrasynthese (Lyon Nord, France). 1-Palmitoyl-2-oleoylphosphatidylcholine (POPC), 1-palmitoyl-2-oleoylphosphatidylethanolamine (POPE), and 1-stearoyl-2-oleoylphosphatidylserine (SOPS) were obtained from Avanti Polar Lipids (Delfzijl, The Netherlands). 2,20-Azobis(2-amidinopropane) dihydrochloride (AAPH), 2,2-diphenyl-1-picryl hydrazyl radical (DPPH), and cholesterol were purchased from Sigma-Aldrich (St Louis, MO, USA). The probe 3-[p-(6-phenyl)-1,3,5-hexatrienyl]propionic acid (DPH-PA) was obtained from Molecular Probes (Eugene, OR, USA).

### 3.2. Liposome Preparation

The study was conducted using liposome membranes whose lipid composition imitated the membrane of tumour cells. This cancer mimic membrane (MM) consisted of 48 mol% POPC, 24 mol% POPE, 8 mol% SOPS, and 20 mol% cholesterol. These lipids were dissolved in chloroform (100 mg/mL), then evaporated to dryness under nitrogen and under vacuum conditions for 90 min. Thereafter, the phosphate buffer (pH 7.4) was added and liposomes were formed by mechanical shaking using vortex until a homogeneous suspension of vesicles was obtained. Subsequently, this suspension was sonicated for 15 min in 0 °C using a 20 kHz sonicator Sonic (Milano, Italia). Liposomes, which were prepared according to the presented procedure in a final concentration equal to 0.1 mg/mL, were used as a mimic bilayer structure in fluorimetric studies. 

### 3.3. Liposome Oxidation Assay

Antioxidant activities of AA were determined using the fluorimetric method described previously by Strugała et. al. [41] with minor modifications. The liposomes at a concentration of 0.1 mg/mL in phosphate buffer (pH 7.4) were incubated for 30 min in darkness with added DPH-PA probe at a concentration of 1 µM. The oxidation reaction was started just before the measurement with AAPH^●^ at the concentration of 1M (dissolved in water) and in the presence of the tested AA in different concentration in the range 0.5–6 μM. A fluorimeter was used to perform measurements (Cary Eclipse, Varian, San Diego, CA, USA). The wavelengths of excitation and emission for the probe were λ_ex_ = 355 nm, λ_em_ = 430 nm as follow. 

The oxidation process of liposomes was initiated with AAPH, which has the ability to decompose at 37 °C, resulting in the appearance of alkyl radicals that oxidase lipids. These radicals react with the DPH-PA probe, whose oxidation causes changes in probe fluorescence, decreasing the fluorescence intensity. A measure of lipid-membrane oxidation was the calculated value of the relative fluorescence intensity of the DPH-PA probe after 30 min. It can be expressed as the F/F_0_ ratio, which is defined as the ratio of the fluorescence of the test sample to the initial fluorescence of the probe [42]. The antioxidant activity of each compound in relation to the inhibition of lipid oxidation was calculated from the equation:

(1) $$\%\;\mathrm{inhibition}=\frac{\left(F_{s}-F_{c}\right)}{\left(F_{b}-F_{c}\right)}\times 100\%$$

where F_s_ denotes the relative fluorescence of the probe oxidized by AAPH^●^ in the presence of AA; F_c_ the relative fluorescence of control sample oxidized by AAPH^●^ in the absence of the AA F_b_, the relative fluorescence of the blank sample (not oxidized by AAPH^●^ and without AA). All measurements were performed for three independent preparations (*n* = 3).

### 3.4. Free-Radical Scavenging Assay

The anti-free radical capacity of AA was determined by the percentage reduction of 2,2-diphenyl-1-picrylhydrazyl radical (DPPH**^●^**) radical concentration. The effect of the studied AA was measured using spectroscopy as described previously by Brand-Williams et al. [43]. DPPH^●^ methanol solutions was mixed with the AA in different final concentrations depending on the tested compound (5 − 60 µM) and placed in a spectrophotometer (Carry 300 Bio, Varian, Santa Clara, CA, USA). The reduction of DPPH^●^ in the sample after 20 min incubation at room temperature with the AA was calculated using the equation:

(2) $$\%\;\mathrm{reduction}\;\mathrm{DPPH}\;\;=\frac{{\Delta A}_{0}-{\Delta A}_{1}}{{\Delta A}_{0}}\times 100\%$$

where ΔA_0_ denotes the change in absorbance at λ = 517 nm after 20 min in the absence of the AA, and ΔA_1_ the change in absorbance at λ = 517 nm after 20 min in the presence of the AA. All determinations were performed in five independent replicates (*n* = 5).

### 3.5. Computational Studies

The electronic structure calculations were carried out in Gaussian16 (rev.C.01) quantum chemistry software package [44]. Geometry optimizations and frequency computations were performed in a framework of density functional theory, namely M06-2X functional [45] conjugated with 6-311+G(d,p) basis set [46,47,48,49]. In the case of the open shell systems, unrestricted calculations were performed. Absence of imaginary frequencies has been confirmed at each stage of the computations, identifying local minima. The choice of this global hybrid exchange–correlation GGA functional was dictated by its particularly good performance in establishing electronic and thermochemical properties of radical species, as evidenced by a recent benchmark study [50] and a number of original papers [51,52,53,54]. Moreover, it can be used to establish acid dissociations constants of the studied compounds in the fitted parameters method [55], which predicts them with a little deviation from experimental ones. To mimic physiologically relevant media, aquatic and lipid, water and pentyl ethanoate were modeled by the solvation model based on density (SMD) [56].

Having regard to the acidic character of aromatic hydroxyl groups, it was necessary to establish a molar fraction of each species in physiological pH. To account for that, the aforementioned fitted parameters method was applied. Only species with non-negligible populations were submitted for the latter studies. Because deprotonations cannot occur in lipids, it was not considered in pentyl ethanoate computations.

The electronic structure was investigated thoroughly in several ways. Firstly, the intrinsic reactivity indices, bond dissociation enthalpy, and ionization potential were estimated in an adiabatic framework. Although this methodology does not account for a radical scavenged, it allows to concentrate on the structure–activity relationship of isolated species, pinpointing motifs with the greatest impact on the radical scavenging potential.

Chemical potential (μ, Equation (3)), chemical hardness (η, Equation (4)) and electrophilicity (ω, Equation (5)), the indices required to construct the eH-DAMA were computed from vertical electron affinities and ionization potentials.

(3) $$\mu =-\frac{\mathrm{vIP}+\mathrm{vEA}}{2}$$

(4) $$\eta =\frac{vIP-vEA}{2}$$

(5) $$\omega =\frac{\mu^{2}}{2\eta }$$

Thermochemical computations were focused on SET and *f*–HAT mechanisms, which are primarily responsible for antioxidative activity. All sites likely to act as H-donors, namely all undissociated aromatic hydroxyl groups, were considered for this purpose. Recent reports, including one dedicated to anthocyanidins, demonstrate that the Bell–Evans–Polanyi principle is valid for the hydrogen atom transfer, thereby allowing us to assume that a hydroxyl group with the lowest Gibbs free energy is at the same time the one that would react most readily with a radical species [57,58,59]. 

### 3.6. Statistical Analysis

Data are shown as mean values ± standard deviation (SD). Statistical analysis was performed using the program Statistica 12.0 (StatSoft, Kraków, Poland). The results were analyzed by one-way ANOVA followed by Duncan test. *p* values < 0.05 were considered statistically significant.

## 4. Conclusions

The experimental studies of antioxidant activity in relation to model lipid membranes showed an extremely high activity of anthocyanidins, while the sequence of this activity did not increase in line with the increasing number of -OH groups in the B ring of the molecule and the activity of their glucoside derivatives was higher than anthocyanidins. Moreover, computational studies highlight that this difference does not necessarily stem from the thermochemical aspect and instead may be ruled by the multiplicity of reactions that antioxidants can undergo in direct interaction with the already oxidized DPH-PA. In both cases, in order to explain properly the AAPH^●^ outcomes, further studies focused on the kinetic reaction supported by molecular dynamics simulations are required. The results obtained in the DPPH^●^ test in both groups of anthocyanins, i.e., groups of anthocyanidins and their glucoside derivatives, showed different sequences of the anti-radical activity, a fact which indicates a complex way of explaining the antioxidant activity of these compounds in relation to the lipid mimic membranes of tumor cells. It was also proven that DPPH^●^ can be readily scavenged not only by means of hydrogen donation, but also through electron transfer, which, in contrast, is not feasible in the environment of the non-polar lipid bilayer. It is believed that, in addition to the high redox potential of the antioxidant molecule, the membrane protection is related to the strategic distribution of anthocyanin molecules in the membrane and their physicochemical properties. More detailed studies of the interaction between anthocyanins and biological membranes are needed in order to better understand this phenomenon.

## Figures and Tables

**Table 1:** The chemical structure of the compounds studied.

Compound	Abbreviations	R1	R2	R3
Peonidin	Pn	-OH	-OCH3	-H
Peonidin 3-*O*-glucoside	Pn 3-glc	Glc	-OCH3	-H
Petunidin	Pt	-OH	-OH	-OCH3
Petunidin 3-*O*-glucoside	Pt 3-glc	Glc	-OH	-OCH3
Delphinidin	Dp	-OH	-OH	-OH
Delphinidin 3-*O*-glucoside	Dp 3-glc	Glc	-OH	-OH

**Table 2:** Dissociation constants (*pK*_a_) and molar fractions (^M^*f*) in %, of the species of each compound under investigation, at pH = 7.4.

	p*K*_a1_	p*K*_a2_	p*K*_a3_	p*K*_a4_	p*K*_a5_	p*K*_a6_	^M^ *f_+_*	^M^ *f_0_*	^M^ *f*_−_	^M^ *f_2_*_−_	^M^ *f_3_*_−_	^M^ *f_4_*_−_	^M^ *f_5_*_−_
Dp	3.55	7.29	7.94	9.39	12.42	14.03	0.01	37.54	48.36	13.95	0.14	0.14	0.00
Dp 3-glc	3.64	6.27	8.34	12.02	13.21	-	0.00	6.23	84.11	9.66	0.00	0.00	-
Pn	4.94	7.11	8.43	9.80	-	-	0.11	31.71	62.30	5.86	0.02	-	-
Pn 3-glc	5.12	6.54	8.42	-	-	-	0.06	11.18	81.02	7.74	-	-	-
Pt	4.88	6.84	8.29	9.07	11.93	-	0.06	19.63	71.06	9.05	0.19	0.00	-
Pt 3-glc	5.14	7.28	7.85	10.98	-	-	0.20	35.82	47.22	16.76	0.00	-	-

**Table 3:** Intrinsic reactivity indices (in kcal mol**^−1^**) of the species with non-negligible populations of the investigated compounds; the lowest values are highlighted by bold type.

		Pentyl Ethanoate		Water									
				+		0		−		2−		3−	
		BDE	IP	BDE	IP	BDE	IP	BDE	IP	BDE	IP	BDE	IP
Dp	C3	77.4	152.0			70.2	96.6		82.6		74.1		67.4
	C3′	78.5				75.8		71.7		66.4		62.3	
	C4′	75.4											
	C5	81.3				74.5		64.2		61.6			
	C5′	82.0				75.4		70.2		66.9		63.0	
	C7	83.6				74.1		63.7					
Dp 3-glc	C3	*-Glu*	152.2			*-Glu*	96.7	*-Glc*	89.2	*-Glc*	83.7		
	C3′	82.4				74.5		70.7		68.4			
	C4′	75.0											
	C5	77.7				73.6							
	C5′	78.9				73.7		71.1		68.6			
	C7	84.3				74.9		74.6					
Pn	C3	74.8	154.3	78.4	118.8	71.4	102.2	66.9	96.1		75.7		
	C4′	81.9		80.6		78.4		75.8		61.3			
	C5	79.7		82.4									
	C7	85.0		87.1		83.1							
Pn 3-glc	C3	*-Glu*	152.4			*-Glc*	103.8	*-Glc*	96.0	*-Glc*	84.9		
	C4′	77.2				78.0		76.0					
	C5	79.7				84.1							
	C7	82.2											
Pt	C3	75.1	153.5			71.6	103.0	67.7	96.8	66.3	87.4		73.1
	C3′	77.8				79.2		78.2					
	C4′	77.6				77.0		75.0		66.4		61.5	
	C5	79.8											
	C7	85.7				83.0							
Pt 3-glc	C3	*-Glu*	151.1	*-Glc*	116.6	*-Glc*	95.0	*-Glc*	85.5	*-Glc*	81.9		
	C3′	77.9		80.8		72.2		67.3		64.7			
	C4′	76.2		76.2									
	C5	83.4		84.0		75.0		71.4					
	C7	84.0		85.9		73.2							

**Table 4:** Gibbs free energies (in kcal mol^−1^) of *f*-HAT reactions between the anthocyanidins’ and anthocyanins’ species and DPPH^●^ in water; the lowest values are bold typed.

		+	0	−	2−	3−
Dp	C3		−2.0			
	C3′		3.6	−0.5	−5.8	−10.0
	C4′					
	C5		2.3	−8.0	−10.6	
	C5′		3.2	−2.1	−5.3	−9.2
	C7		1.9	−8.5		
Dp 3-glc	C3	*-Glc*	*-Glc*	*-Glc*	*-Glc*	*-Glc*
	C3′		2.3	−1.5	−3.8	
	C4′					
	C5		1.4			
	C5′		1.5	−1.1	−3.6	
	C7		2.7	2.4		
Pn	C3	6.2	−0.9	−5.3		
	C4′	8.4	6.2	3.6	−10.9	
	C5	10.1	10.9			
	C7	14.8				
Pn 3-glc	C3	*-Glc*	*-Glc*	*-Glc*	*-Glc*	*-Glc*
	C4′		5.8	3.8		
	C5		11.9			
	C7					
Pt	C3		−0.6	−4.5	−5.9	
	C3′		6.9	5.9		
	C4′		4.7	2.8	−5.8	−10.7
	C5					
	C7		10.8			
Pt 3-glc	C3	*-Glc*	*-Glc*	*-Glc*	*-Glc*	*-Glc*
	C3′	8.5	0.0	−4.9	−7.5	
	C4′	4.0				
	C5	11.7	2.8	−0.9		
	C7	13.6	1.0			

**Table 5:** Activation energies (ΔG^≠^, in kcal mol^−1^) and kinetic constants (k, in M^−1^ s^−1^) of SET reactions between the anthocyanidins’ and the anthocyanins’ species and DPPH^●^ in water.

	+		0		1−		2−		3−	
	ΔG^≠^	*k*	ΔG^≠^	*k*	ΔG^≠^	*k*	ΔG^≠^	*k*	ΔG^≠^	*k*
**Dp**			12.1	8.3 × 10^3^	3.4	5.4 × 10^9^	0.8	7.4 × 10^9^	0.0	7.5 × 10^9^
**Dp 3-glc**			12.3	6.0 × 10^3^	6.8	6.5 × 10^7^	3.8	4.3 × 10^9^		
**Pn**	39.2	1.1 × 10^−16^	17.7	7.0 × 10^−1^	11.6	1.9 × 10^4^	0.7	7.5 × 10^9^		
**Pn 3-glc**			19.3	4.2 × 10^−2^	11.5	2.12 × 10^4^	4.2	3.1 × 10^9^		
**Pt**			18.8	1.1 × 10^−1^	12.2	6.8 × 10^3^	5.6	4.6 × 10^8^	0.4	2.0 × 10^9^
**Pt 3-glc**	35.6	4.7 × 10^−14^	10.9	6.0 × 10^4^	4.7	1.7 × 10^9^	3.3	4.8 × 10^9^		

## Data Availability

Not applicable.

## Supplementary Materials

The following supporting information can be downloaded at: https://www.mdpi.com/article/10.3390/ijms23105432/s1.
