# Supplementary material for: Analytical and Theoretical Studies of Antioxidant Properties of Chosen Anthocyanins; A Structure-Dependent Relationships
Source: Int J Mol Sci. 2022 May 12;23(10):5432. doi: 10.3390/ijms23105432 (PMC9141991; doi:10.3390/ijms23105432)
Supplement: Supplementary file 1 [file ijms-23-05432-s001.zip › ijms-1713110-supplementary.pdf]

SUPPLEMENTARY MATERIALS

Figure S1. Distribution diagrams with deprotonation routes of the (**a**) delphinidin; (**b**) delphinidin 3-*O*-glucoside; (**c**) peonidin; (**d**) peonidin 3-*O*-glucoside; (**e**) petunidin; (**f**) petunidin3-*O*-glucoside.

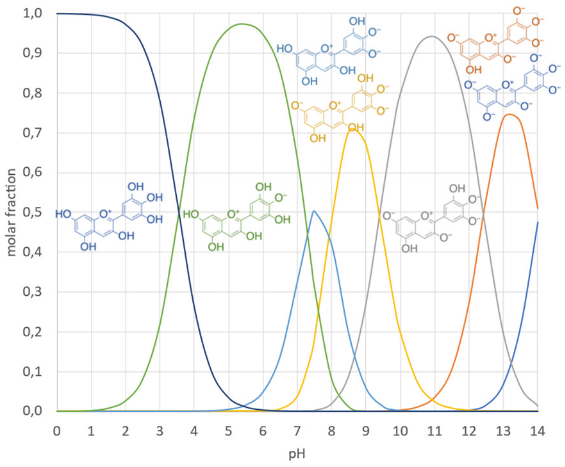

(**a**)

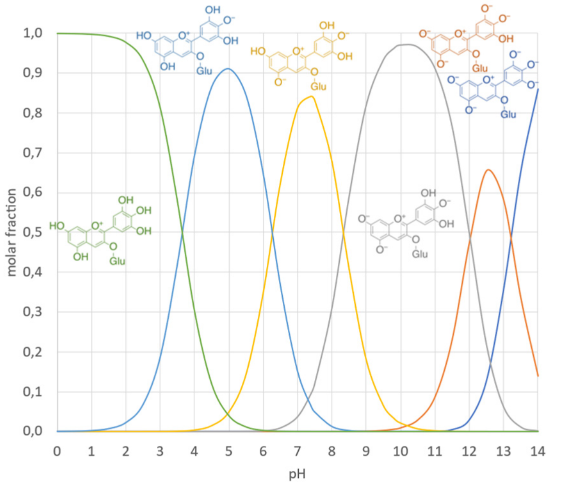

(**b**)

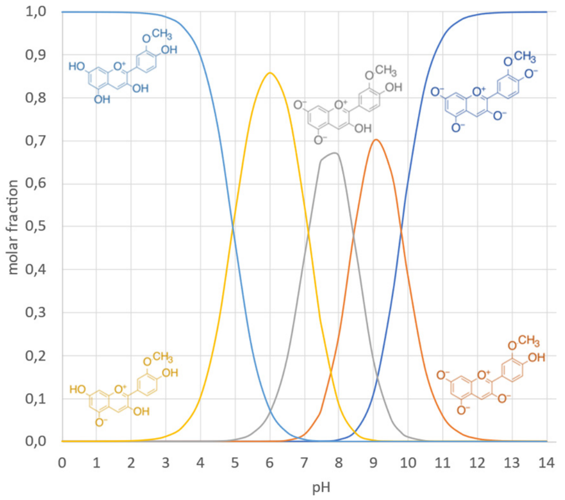

(**c**)

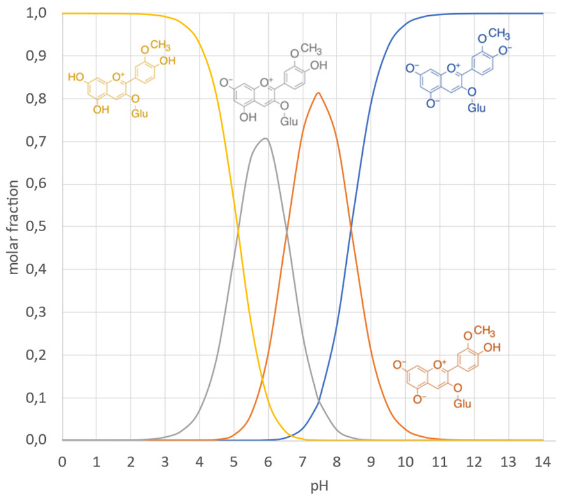

(**d**)

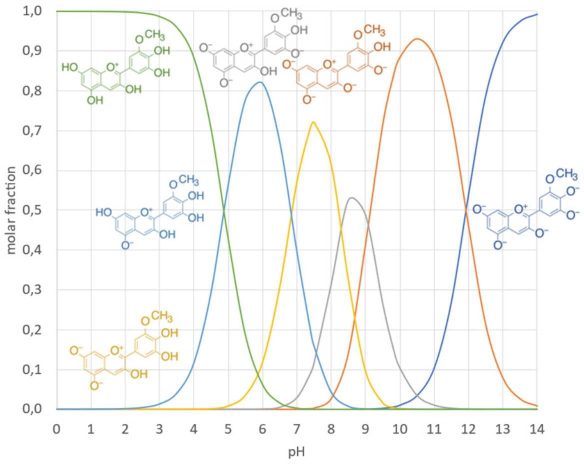

(**e**)

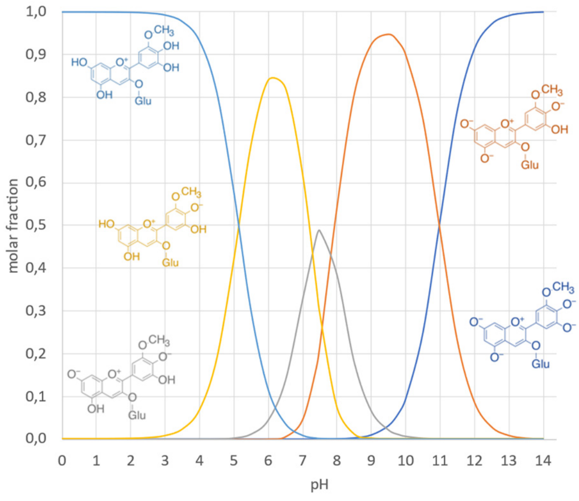

(**f**)

Table S1. Proton affinities (kcal mol^{-1}) of anthocyanidins and anthocyanins investigated along with the reaction site. The lowest values are underlined.

		1+	0	1–	2–	3–	4-
Dp	C3	25.8	**29.1**				
	C3’	26.4	37.2	39.8	45.4	**45.2**	
	C4’	**17.3**					
	C5	22.6	39.1	32.4	**35.7**		
	C5’	32.1	29.7	41.4	46.4	45.6	**50.3**
	C7	22.4	29.3	**31.2**			
Dp 3-glc	C3	*-Glu*	*-Glu*	*-Glu*	*-Glu*	*-Glu*	*-Glu*
	C3’	33.5	37.3	40.1	**44.0**		
	C4'	**17.6**					
	C5	21.4	**25.9**				
	C5’	29.2	38.0	41.4	44.6	**47.7**	
	C7	25.8	29.0	**32.4**			
Pn	C3	23.7	29.0	**32.7**			
	C4’	26.4	30.7	33.8	**37.0**		
	C5	**21.7**					
	C7	23.4	**28.5**				
Pn 3-glc	C3	*-Glu*	*-Glu*	*-Glu*	*-Glu*		
	C4’	22.8	30.1	**32.7**			
	C5	23.9	**26.7**				
	C7	**22.3**					
Pt	C3	23.2	28.7	33.3	**34.7**		
	C3’	28.8	30.3	**32.3**			
	C4’	24.2	28.3	32.6	41.3	**43.7**	
	C5	**21.5**					
	C7	22.7	**27.7**				
Pt-3-glc	C3	*-Glu*	*-Glu*	*-Glu*	*-Glu*	*-Glu*	
	C3’	29.8	35.6	37.9	**40.7**		
	C4’	**22.4**					
	C5	23.7	**29.1**	**30.9**			
	C7	23.5	**29.1**				

Table S2. Dipole momenta (μ, in D) and their corresponding vector in 3D-dimensions, obtained for the aglycones and their glycosides in pentyl ethanoate (cation form) and water (neutral form).

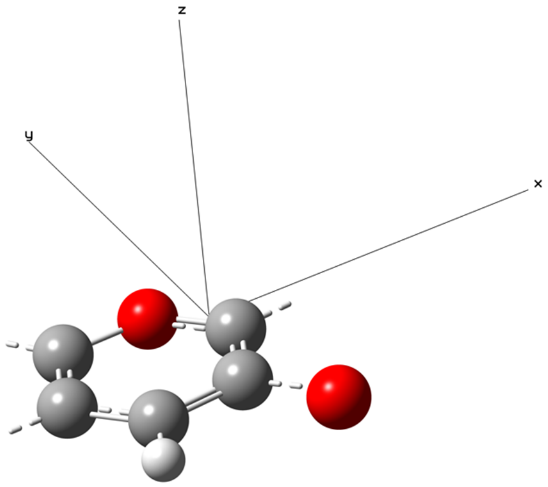

	pentyl ethanoate		water	
	+		0	
	μ [D]	vector (x,y,z)	μ [D]	(x,y,z)
Dp	10.2	(-10.1, 0.3, 1.1)	22.5 (6)	(-21.9, -5.3, 0)
Dp 3-glc	6.8	(-0.0, 5.4, -4.1)	14.1 (2)	(-11.7, 6.3, -4.9)
Pn	2.6	(1.7, 1.7, 1.1)	15.5 (4)	(-13.4, 7.7, 0.3)
Pn-3-glc	12.0	(-11.6, -2.6, 1.9)	11.2 (1)	(-9.8, -4.8, -2.6)
Pt	4.7	(-3.4, 3.2, -0.5)	15.3 (3)	(11.3, 10.3, 0.8)
Pt 3-glc	10.6	(7.6, 7.2, -2.0)	19.5 (5)	(-7.5, 17.9, -1.9)

Table S3. The reactivity of the studied anthocyanidins and their glycosides in SET and HAT mechanisms towards common radicals in water.

Delphinidin

Neutral

R-OH + OH* -> R-OH+* + OH-	[]	R-OH + OOH* -> R-OH+* + OOH-	[kcal/mol]
adiabatic	-9,2	adiabatic	14,2
vertical	-5,1	vertical	27,5
activation	1,6	activation	14,2
rate constants	8.37E+9	rate constants	2.36E+02
R-OH + OH* -> R-O* + H2O		R-OH + OOH* -> R-O* + H2O2	
**C3**	-45,4	**C3**	-7,7
**C3'**	-39,8	**C3'**	-2,2
**C5**	-41,1	**C5**	-3,4
**C5'**	-40,2	**C5'**	-2,5
**C7**	-41,5	**C7**	-3,9
R-OH + CH3O -> R-OH+* + CH3O-*	[kcal/mol]		
adiabatic	10,8		
vertical	17,7		
activation	11,4		
rate constants	2,96E+04		
R-OH + CH3O -> R-O* + CH3OH*			
**C3**	-27,2		
**C3'**	-21,6		
**C5**	-22,9		
**C5'**	-21,9		
**C7**	-23,3		
R-OH + SH -> R-OH+* + SH-*	[kcal/mol]		
adiabatic	6,4		
vertical	11,2		
activation	6,5		
rate constants	1,01E+08		
R-OH + SH -> R-O* + H2S*			
**C3**	-31,0		
**C3'**	-25,4		
**C5**	-26,7		
**C5'**	-25,7		
**C7**	-27,1		

Anion

R-OH + OH* -> R-OH+* + OH-	[kcal/mol]	R-OH + OOH* -> R-OH+* + OOH-	[kcal/mol]
adiabatic	-23,1	adiabatic	0,2
vertical	-17,1	vertical	15,6
activation	12,2	activation	4,0
rate constants	7,27E+03	rate constants	3.99E+09
R-OH + OH* -> R-O* + H2O		R-OH + OOH* -> R-O* + H2O2	
**C3'**	-43,9	**C3'**	-6,3
**C5**	-51,4	**C5**	-13,7
**C5'**	-45,4	**C5'**	-7,8
**C7**	-51,9	**C7**	-14,2
R-OH + CH3O* -> R-OH+* + CH3O-	[kcal/mol]		
adiabatic	-3,1		
vertical	5,7		
activation	0,9		
rate constants	7.85E+9		
R-OH + CH3O* -> R-O* + CH3OH			
**C3'**	-25,7		
**C5**	-33,2		
**C5'**	-27,2		
**C7**	-33,7		
R-OH + SH* -> R-OH+* + SH-	[kcal/mol]		
adiabatic	-7,5		
vertical	-0,7		
activation	0,0		
rate constants	8.52E+9		
R-OH + SH* -> R-O* + H2S			
**C3'**	-29,5		
**C5**	-37,0		
**C5'**	-31,0		
**C7**	-37,5		

Dianion

R-OH + OH* -> R-OH+* + OH-	[kcal/mol]	R-OH + OOH* -> R-OH+* + OOH-	[kcal/mol]
adiabatic	-31,6	*adiabatic*	-8,3
vertical	-25,0	*vertical*	7,7
activation	23,7	*activation*	0,9
rate constants	2,74E-05	*rate constants*	8.03E+9
R-OH + OH* -> R-O* + H2O		R-OH + OOH* -> R-O* + H2O2	
**C3'**	-49,2	**C3'**	-11,5
**C5**	-54,0	**C5**	-16,4
**C5'**	-48,7	**C5'**	-11,0
R-OH + CH3O* -> R-OH+* + CH3O-	[kcal/mol]		
adiabatic	-11,7		
vertical	-2,2		
activation	0,1		
rate constants	7.92E+9		
R-OH + CH3O* -> R-O* + CH3OH			
**C3'**	-31,0		
**C5**	-35,8		
**C5'**	-30,5		
R-OH + SH* -> R-OH+* + SH-	[kcal/mol]		
adiabatic	-16,1		
vertical	-8,6		
activation	2,5		
rate constants	7.84E+9		
R-OH + SH* -> R-O* + H2S			
**C3'**	-34,8		
**C5**	-39,6		
**C5'**	-34,3		

Trianion

R-OH + OH -> R-OH+* + OH-*	[kcal/mol]	*R-OH + OOH* -> R-OH+* + OOH-*	[kcal/mol]
adiabatic	-38,3	*adiabatic*	-15,0
vertical	-31,2	*vertical*	1,5
activation	34,8	*activation*	0,0
rate constants	2,03E-13	*rate constants*	8.07E+9
R-OH + OH -> R-O* + H2O*		*R-OH + OOH* -> R-O* + H2O2*	
**C3'**	-53,3	**C3'**	-15,7
**C5'**	-52,6	**C5'**	-15,0
R-OH + CH3O -> R-OH+* + CH3O-*	[kcal/mol]		
adiabatic	-18,4		
vertical	-8,4		
activation	-0,7		
rate constants	7.74E+9		
R-OH + CH3O -> R-O* + CH3OH*			
**C3'**	-35,1		
**C5'**	-34,4		
R-OH + SH -> R-OH+* + SH-*	[kcal/mol]		
adiabatic	-22,8		
vertical	-14,8		
activation	6,9		
rate constants	5,96E+07		
R-OH + SH -> R-O* + H2S*			
**C3'**	-38,9		
**C5'**	-38,2		

Delphinidin 3-*O*-glucoside

neutral

R-OH + OH -> R-OH+* + OH-* [kcal/mol]	*R-OH + OOH* -> R-OH+* + OOH-* [kcal/mol]
adiabatic -9,0	*adiabatic* 14,3
vertical -4,9	*vertical* 27,8
activation 1,5	*activation* 14,3
rate constants 8.93+09	*rate constants* 2,00E+02
R-OH + OH -> R-O* + H2O*	*R-OH + OOH* -> R-O* + H2O2*
**C3'** -41,1	**C3'** -3,4
**C5** -42,0	**C5** -4,3
**C5'** -41,9	**C5'** -4,2
**C7** -40,7	**C7** -3,0
R-OH + CH3O -> R-OH+* + CH3O-* [kcal/mol]	
adiabatic 10,9	
vertical 17,9	
activation 11,4	
rate constants 2,54E+04	
R-OH + CH3O -> R-O* + CH3OH*	
**C3'** -22,8	
**C5** -23,8	
**C5'** -23,7	
**C7** -22,4	
R-OH + SH -> R-OH+* + SH-* [kcal/mol]	
adiabatic 6,6	
vertical 11,5	
activation 6,8	
rate constants 6.97+7	
R-OH + SH -> R-O* + H2S*	
**C3'** -26,6	
**C5** -27,6	
**C5'** -27,5	
**C7** -26,2	

Anion

R-OH + OH -> R-OH+* + OH-*	[kcal/mol]
adiabatic	-16,5
vertical	-11,3
activation	6,1
rate constants	1,92E+08
R-OH + OH -> R-O* + H2O*	
**C3'**	-44,9
**C5'**	-44,5
**C7**	-41,0

R-OH + OOH -> R-OH+* + OOH-*	[kcal/mol]
adiabatic	6,8
vertical	21,4
activation	7,8
rate constants	1,11E+07
R-OH + OOH -> R-O* + H2O2*	
**C3'**	-7,3
**C5'**	-6,8
**C7**	-3,3

R-OH + CH3O -> R-OH+* + CH3O-*	[kcal/mol]
adiabatic	3,5
vertical	11,5
activation	4,1
rate constants	3.42E+09
R-OH + CH3O -> R-O* + CH3OH*	
**C3'**	-26,7
**C5'**	-26,3
**C7**	-22,8

R-OH + SH -> R-OH+* + SH-*	[kcal/mol]
adiabatic	-0,9
vertical	5,1
activation	1,08
rate constants	9,01E+9
R-OH + SH -> R-O* + H2S*	
**C3'**	-30,5
**C5'**	-30,1
**C7**	-26,6

Dianion

R-OH + OH -> R-OH+* + OH-*	[kcal/mol]	*R-OH + OOH* -> R-OH+* + OOH-*	[kcal/mol]
adiabatic	-22,1	*adiabatic*	1,3
vertical	-16,4	*vertical*	16,2
activation	11,8	*activation*	4,4
rate constants	1,40E+04	*rate constants*	2.75E+09
R-OH + OH -> R-O* + H2O*		*R-OH + OOH* -> R-O* + H2O2*	
**C3'**	-47,2	**C3'**	-9,5
**C5'**	-47,0	**C5'**	-9,3
R-OH + CH3O -> R-OH+* + CH3O-*	[kcal/mol]		
adiabatic	-2,1		
vertical	6,4		
activation	1,20		
rate constants	8,22E+9		
R-OH + CH3O -> R-O* + CH3OH*			
**C3'**	-28,9		
**C5'**	-28,7		
R-OH + SH -> R-OH+* + SH-*	[kcal/mol]		
adiabatic	-6,5		
vertical	-0,1		
activation	0,0		
rate constants	9.12E+9		
R-OH + SH -> R-O* + H2S*			
**C3'**	-32,8		
**C5'**	-32,5		

Peonidin

Cation

R-OH + OH -> R-OH+* + OH-*	[kcal/mol]
adiabatic	13,0
vertical	17,5
activation	17,0
rate constants	2,09E+00
R-OH + OH -> R-O* + H2O*	
**C3**	-37,2
**C4'**	-35,0
**C5**	-33,3
**C7**	-28,5

R-OH + OOH -> R-OH+* + OOH-*	[kcal/mol]
adiabatic	36,4
vertical	50,2
activation	45,7
rate constants	2,12E-21
R-OH + OOH -> R-O* + H2O2*	
**C3**	0,5
**C4'**	2,7
**C5**	4,4
**C7**	9,1

R-OH + CH3O -> R-OH+* + CH3O-*	[kcal/mol]
adiabatic	33,0
vertical	40,3
activation	55,6
rate constants	1,05E-28
R-OH + CH3O -> R-O* + CH3OH*	
**C3**	-19,0
**C4'**	-16,8
**C5**	-15,0
**C7**	-10,3

R-OH + SH -> R-OH+* + SH-*	[kcal/mol]
adiabatic	28,6
vertical	33,9
activation	54,21
rate constants	1,13E-27
R-OH + SH -> R-O* + H2S*	
**C3**	-22,8
**C4'**	-20,6
**C5**	-18,8
**C7**	-14,1

Neutral

R-OH + OH* -> R-OH+* + OH-	[kcal/mol]
adiabatic	-3,6
vertical	-0,7
activation	0,0
rate constants	8.48E+09
R-OH + OH -> R-O* + H2O*	
**C3**	-44,2
**C4'**	-37,2
**C7**	-32,5

R-OH + OOH* -> R-OH+* + OOH-	[kcal/mol]
adiabatic	19,8
vertical	32,0
activation	21,0
rate constants	2,57E-03
R-OH + OOH -> R-O* + H2O2*	
**C3**	-6,6
**C4'**	0,4
**C7**	5,1

R-OH + CH3O* -> R-OH+* + CH3O-	[kcal/mol]
adiabatic	16,4
vertical	22,1
activation	21,4
rate constants	1,23E-03
R-OH + CH3O -> R-O* + CH3OH*	
**C3**	-26,0
**C4'**	-19,0
**C7**	-14,3

R-OH + SH* -> R-OH+* + SH-	[kcal/mol]
adiabatic	12,0
vertical	15,7
activation	16,65
rate constants	3,84E+00
R-OH + SH -> R-O* + H2S*	
**C3**	-29,8
**C4'**	-22,8
**C7**	-18,1

Anion

R-OH + OH -> R-OH+* + OH-*	[kcal/mol]
adiabatic	-9,6
vertical	-7,0
activation	4,7100
rate constants	1,74E+09
R-OH + OH -> R-O* + H2O*	
**C3**	-48,7
**C4'**	-39,8

R-OH + OOH -> R-OH+* + OOH-*	[kcal/mol]
adiabatic	13,7
vertical	25,6
activation	13,7700
rate constants	5,01E+02
R-OH + OOH -> R-O* + H2O2*	
**C3**	-11,1
**C4'**	-2,1

R-OH + CH3O -> R-OH+* + CH3O-*	[kcal/mol]
adiabatic	10,3
vertical	15,8
activation	11,3500
rate constants	2,98E+04
R-OH + CH3O -> R-O* + CH3OH*	
**C3**	-30,5
**C4'**	-21,5

R-OH + SH -> R-OH+* + SH-*	[kcal/mol]
adiabatic	5,9
vertical	9,3
activation	6,4
rate constants	1,35E+08
R-OH + SH -> R-O* + H2S*	
**C3**	-34,3
**C4'**	-25,3

Dianion

R-OH + OH -> R-OH+* + OH-* [kcal/mol]<br>*adiabatic* -30,0<br>*vertical* -26,2<br>*activation* 45,16<br>*rate constants* 4,86E-21<br>*R-OH + OH* -> R-O* + H2O*<br>**C4'** -54,3	*R-OH + OOH* -> R-OH+* + OOH-* [kcal/mol]<br>*adiabatic* -6,7<br>*vertical* 6,5<br>*activation* 0,80<br>*rate constants* 8.04E+09<br>*R-OH + OOH* -> R-O* + H2O2*<br>**C4'** -16,6
R-OH + CH3O -> R-OH+* + CH3O-* [kcal/mol]<br>*adiabatic* -10,0<br>*vertical* -3,4<br>*activation* 0,44<br>*rate constants* 7.91E+09<br>*R-OH + CH3O* -> R-O* + CH3OH*<br>**C4'** -36,1	
R-OH + SH -> R-OH+* + SH-* [kcal/mol]<br>*adiabatic* -14,4<br>*vertical* -9,8<br>*activation* 5,22<br>*rate constants* 8.35E+08<br>*R-OH + SH* -> R-O* + H2S*<br>**C4'** -39,9	

Peonidin 3-*O*-glucoside

Neutral

R-OH + OH -> R-OH+* + OH-*	[kcal/mol]	*R-OH + OOH* -> R-OH+* + OOH-*	[kcal/mol]
adiabatic	-1,9	*adiabatic*	21,4
vertical	2,9	*vertical*	35,6
activation	0,4	*activation*	22,3
rate constants	9.09E+09	*rate constants*	2,73E-04
R-OH + OH -> R-O* + H2O*		*R-OH + OOH* -> R-O* + H2O2*	
**C4'**	-37,6	**C4'**	0,1
**C5**	-31,5	**C5**	6,2
R-OH + CH3O -> R-OH+* + CH3O-*	[kcal/mol]		
adiabatic	18,1		
vertical	25,7		
activation	21,7		
rate constants	7,34E-04		
R-OH + CH3O -> R-O* + CH3OH*			
**C4'**	-19,3		
**C5**	-13,3		
R-OH + SH -> R-OH+* + SH-*	[kcal/mol]		
adiabatic	13,7		
vertical	19,3		
activation	16,63		
rate constants	4,01E+00		
R-OH + SH -> R-O* + H2S*			
**C4'**	-23,2		
**C5**	-17,1		

Anion

R-OH + OH -> R-OH+* + OH-*	[kcal/mol]	*R-OH + OOH* -> R-OH+* + OOH-*	[kcal/mol]
adiabatic	-9,8	*adiabatic*	13,5
vertical	-6,1	*vertical*	26,6
activation	2,5100	*activation*	13,5000
rate constants	8,25E+9	*rate constants*	7,84E+02
R-OH + OH -> R-O* + H2O*		*R-OH + OOH* -> R-O* + H2O2*	
**C4'**	-39,6	**C4'**	-1,9
R-OH + CH3O -> R-OH+* + CH3O-*	[kcal/mol]		
adiabatic	10,2		
vertical	16,7		
activation	10,7300		
rate constants	8,51E+04		
R-OH + CH3O -> R-O* + CH3OH*			
**C4'**	-21,4		
R-OH + SH -> R-OH+* + SH-*	[kcal/mol]		
adiabatic	5,8		
vertical	10,3		
activation	5,89		
rate constants	2,87E+08		
R-OH + SH -> R-O* + H2S*			
**C4'**	-25,2		

Dianion

R-OH + OH -> R-OH+* + OH-*	[kcal/mol]	*R-OH + OOH* -> R-OH+* + OOH-*	[kcal/mol]
adiabatic	-20,9	*adiabatic*	2,4
vertical	-16,0	*vertical*	16,7
activation	13,0600	*activation*	4,8800
rate constants	1,65E+03	*rate constants*	1,39E+09
R-OH + CH3O -> R-OH+* + CH3O-*	[kcal/mol]		
adiabatic	-0,9		
vertical	6,8		
activation	1,5000		
rate constants	8.21E+9		
R-OH + SH -> R-OH+* + SH-*	[kcal/mol]		
adiabatic	-5,3		
vertical	0,4		
activation	0,0100		
rate constants	9.08E+09		

Petunidin

Neutral

R-OH + OH -> R-OH+* + OH-*	[kcal/mol]	*R-OH + OOH* -> R-OH+* + OOH-*	[kcal/mol]
adiabatic	-2,7	*adiabatic*	20,6
vertical	-0,1	*vertical*	32,5
activation	0,0000	*activation*	22,1900
rate constants	8.45E+09	*rate constants*	3,36E-04
R-OH + OH -> R-O* + H2O*		*R-OH + OOH* -> R-O* + H2O2*	
**C3**	-44,0	**C3**	-6,3
**C3'**	-36,4	**C3'**	1,2
**C4'**	-38,7	**C4'**	-1,0
**C7**	-32,6	**C7**	5,1
R-OH + CH3O -> R-OH+* + CH3O-*	[kcal/mol]		
adiabatic	17,3		
vertical	22,6		
activation	24,0900		
rate constants	1,35E-05		
R-OH + CH3O -> R-O* + CH3OH*			
**C3**	-25,7		
**C3'**	-18,2		
**C4'**	-20,4		
**C7**	-14,4		
R-OH + SH -> R-OH+* + SH-*	[kcal/mol]		
adiabatic	12,9		
vertical	16,2		
activation	19,88		
rate constants	1,65E-02		
R-OH + SH -> R-O* + H2S*			
**C3**	-29,6		
**C3'**	-22,0		
**C4'**	-24,2		
**C7**	-18,2		

Anion

R-OH + OH* -> R-OH+* + OH-	[kcal/mol]
adiabatic	-8,9
vertical	-7,1
activation	7,0000
rate constants	4,58E+07
R-OH + OH* -> R-O* + H2O	
**C3**	-47,9
**C3'**	-37,5
**C4'**	-40,6

R-OH + OOH* -> R-OH+* + OOH-	[kcal/mol]
adiabatic	14,4
vertical	25,6
activation	14,6300
rate constants	1,17E+02
R-OH + OOH* -> R-O* + H2O2	
**C3**	-10,3
**C3'**	0,2
**C4'**	-2,9

R-OH + CH3O* -> R-OH+* + CH3O-	[kcal/mol]
adiabatic	11,1
vertical	15,7
activation	13,4000
rate constants	9,39E+02
R-OH + CH3O* -> R-O* + CH3OH	
**C3**	-29,7
**C3'**	-19,2
**C4'**	-22,4

R-OH + SH* -> R-OH+* + SH-	[kcal/mol]
adiabatic	6,7
vertical	9,3
activation	8,32
rate constants	4,97E+06
R-OH + SH* -> R-O* + H2S	
**C3**	-33,5
**C3'**	-23,0
**C4'**	-26,2

Dianion

R-OH + OH -> R-OH+* + OH-*	[kcal/mol]	*R-OH + OOH* -> R-OH+* + OOH-*	[kcal/mol]
adiabatic	-18,3	*adiabatic*	5,0
vertical	-13,4	*vertical*	19,2
activation	9,1600	*activation*	6,4900
rate constants	1,19E+06	*rate constants*	1,09E+08
R-OH + OH -> R-O* + H2O*		*R-OH + OOH* -> R-O* + H2O2*	
**C3**	-49,3	**C3**	-11,6
**C4'**	-49,2	**C4'**	-11,6
R-OH + CH3O -> R-OH+* + CH3O-*	[kcal/mol]		
adiabatic	1,7		
vertical	9,4		
activation	2,8700		
rate constants	4,90E+10		
R-OH + CH3O -> R-O* + CH3OH*			
**C3**	-31,1		
**C4'**	-31,0		
R-OH + SH -> R-OH+* + SH-*	[kcal/mol]		
adiabatic	-2,7		
vertical	2,9		
activation	0,38		
rate constants	8.44E+09		
R-OH + SH -> R-O* + H2S*			
**C3**	-34,9		
**C4'**	-34,8		

Trianion

R-OH + OH -> R-OH+* + OH-*	[kcal/mol]	*R-OH + OOH* -> R-OH+* + OOH-*	[kcal/mol]
adiabatic	-32,6	*adiabatic*	-9,3
vertical	-27,8	*vertical*	4,9
activation	40,2500	*activation*	0,4200
rate constants	1,93E-17	*rate constants*	8.04E+09
R-OH + OH -> R-O* + H2O*		*R-OH + OOH* -> R-O* + H2O2*	
**C4'**	-54,1	**C4'**	-16,4
R-OH + CH3O -> R-OH+* + CH3O-*	[kcal/mol]		
adiabatic	-12,6		
vertical	-5,0		
activation	0,8200		
rate constants	7.81E+09		
R-OH + CH3O -> R-O* + CH3OH*			
**C4'**	-35,8		
R-OH + SH -> R-OH+* + SH-*	[kcal/mol]		
adiabatic	-17,0		
vertical	-11,4		
activation	5,8		
rate constants	3,47E+08		
R-OH + SH -> R-O* + H2S*			
**C4'**	-39,7		

Petunidin 3-*O*-glucoside

Cation

R-OH + OH -> R-OH+* + OH-*	[kcal/mol]
adiabatic	10,8
vertical	15,7
activation	12,5800
rate constants	3,75E+03
R-OH + OH -> R-O* + H2O*	
**C3'**	-34,9
**C4'**	-39,4
**C5**	-31,6
**C7**	-29,7

R-OH + OOH -> R-OH+* + OOH-*	[kcal/mol]
adiabatic	34,1
vertical	48,3
activation	41,1900
rate constants	3,95E-18
R-OH + OOH -> R-O* + H2O2*	
**C3'**	2,8
**C4'**	-1,7
**C5**	6,0
**C7**	7,9

R-OH + CH3O -> R-OH+* + CH3O-*	[kcal/mol]
adiabatic	30,8
vertical	38,5
activation	48,1300
rate constants	3,26E-23
R-OH + CH3O -> R-O* + CH3OH*	
**C3'**	-16,6
**C4'**	-21,1
**C5**	-13,4
**C7**	-11,5

R-OH + SH -> R-OH+* + SH-*	[kcal/mol]
adiabatic	26,4
vertical	32,0
activation	45,7100
rate constants	1,91E-21
R-OH + SH -> R-O* + H2S*	
**C3'**	-20,4
**C4'**	-25,0
**C5**	-17,2
**C7**	-15,3

Neutral

R-OH + OH -> R-OH+* + OH-*	[kcal/mol]
adiabatic	-10,7
vertical	-5,8
activation	1,7200
rate constants	8.85E+09
R-OH + OH -> R-O* + H2O*	
**C3'**	-43,4
**C5**	-40,6
**C7**	-42,4

R-OH + OOH -> R-OH+* + OOH-*	[kcal/mol]
adiabatic	12,6
vertical	26,8
activation	12,6500
rate constants	3,34E+03
R-OH + OOH -> R-O* + H2O2*	
**C3'**	-5,7
**C5**	-2,9
**C7**	-4,7

R-OH + CH3O -> R-OH+* + CH3O-*	[kcal/mol]
adiabatic	9,2
vertical	16,9
activation	9,2700
rate constants	9,89E+05
R-OH + CH3O -> R-O* + CH3OH*	
**C3'**	-25,1
**C5**	-22,3
**C7**	-24,2

R-OH + SH -> R-OH+* + SH-*	[kcal/mol]
adiabatic	4,8
vertical	10,5
activation	4,84
rate constants	1,48E+09
R-OH + SH -> R-O* + H2S*	
**C3'**	-28,9
**C5**	-26,1
**C7**	-28,0

MonoAnion

R-OH + OH -> R-OH+* + OH-*	[kcal/mol]
adiabatic	-20,2
vertical	-14,8
activation	10,1400
rate constants	2,29E+05
R-OH + OH -> R-O* + H2O*	
**C3'**	-48,3
**C5**	-44,2

R-OH + OOH -> R-OH+* + OOH-*	[kcal/mol]
adiabatic	3,1
vertical	17,9
activation	5,4100
rate constants	6,23E+08
R-OH + OOH -> R-O* + H2O2*	
**C3'**	-10,6
**C5**	-6,6

R-OH + CH3O -> R-OH+* + CH3O-*	[kcal/mol]
adiabatic	-0,2
vertical	8,0
activation	1,9500
rate constants	7.97E+9
R-OH + CH3O -> R-O* + CH3OH*	
**C3'**	-30,1
**C5**	-26,0

R-OH + SH -> R-OH+* + SH-*	[kcal/mol]
adiabatic	-4,6
vertical	1,6
activation	0,1
rate constants	9.07E+09
R-OH + SH -> R-O* + H2S*	
**C3'**	-33,9
**C5**	-29,8

Dianion

R-OH + OH -> R-OH+* + OH-* [kcal/mol]<br>*adiabatic* -23,8<br>*vertical* -17,2<br>*activation* 11,2100<br>*rate constants* 3,79E+04<br>*R-OH + OH* -> R-O* + H2O*<br>**C3'** -50,9	*R-OH + OOH* -> R-OH+* + OOH-* [kcal/mol]<br>*adiabatic* -0,5<br>*vertical* 15,5<br>*activation* 3,7500<br>*rate constants* 4.81E+09<br>*R-OH + OOH* -> R-O* + H2O2*<br>**C3'** -13,2
R-OH + CH3O -> R-OH+* + CH3O-* [kcal/mol]<br>*adiabatic* -3,8<br>*vertical* 5,6<br>*activation* 0,8300<br>*rate constants* 8.21E+09<br>*R-OH + CH3O* -> R-O* + CH3OH*<br>**C3'** -32,7	
R-OH + SH -> R-OH+* + SH-* [kcal/mol]<br>*adiabatic* -8,2<br>*vertical* -0,8<br>*activation* 0,02<br>*rate constants* 9.07E+09<br>*R-OH + SH* -> R-O* + H2S*<br>**C3'** -36,5	
